# Neighborhood Disadvantage, Cognition, and Health Self-Efficacy of Older Adults in a Clinical Population

**DOI:** 10.1093/geroni/igaa057.1563

**Published:** 2020-12-16

**Authors:** Kristen Berg, Nikolas I Krieger, Douglas Einstadter, Lorella Shamakian, Jarrod Dalton, Adam Perzynski

**Affiliations:** 1 Case Western Reserve University at MetroHealth, Cleveland, Ohio, United States; 2 Lerner Research Institute - Cleveland Clinic, Cleveland, Ohio, United States; 3 The MetroHealth System, Cleveland, Ohio, United States; 4 Cleveland Clinic Lerner College of Medicine at Case Western Reserve University, Cleveland, Ohio, United States

## Abstract

The Medicare Annual Wellness Visit (MWV) includes an assessment of health risks for older adults in the United States. Research suggests that neighborhood-level social inequality influences multiple health outcomes. We sought to examine the association between neighborhood socioeconomic position and older adults’ cognition, health management self-efficacy, and other health risks. We identified a cohort of 12,434 adults aged 65 and over from the NEOCARE Learning Health Registry who attended a routine MWV between 2011 and 2019. NEOCARE includes electronic health record and neighborhood data from 1999-2017 on over 3 million unique Northeast Ohio individuals. The study population was 60% White, 32% Black or African American, 64% female, and 90% non-Hispanic. Over 60% were ages 65-74, 29% 75-84, and 10% 85 years or older (range from 65 to 101). We used ANOVA and chi square tests to examine variation in health risks by quintile of census tract area deprivation index. Cognitive functioning differed across quintiles of area deprivation and Bonferroni-corrected tests indicated that adults in the most socioeconomically disadvantaged neighborhoods had lower average cognitive screening scores as compared to older adults in less disadvantaged areas (F=53.50, df=4, n=12,204, p<.001). The proportion of adults feeling efficacious in managing their health differed according to area deprivation, with adults in more disadvantaged neighborhoods having slightly lower self-efficacy, (x2=11.01, df=8, n=11,937, p<.001). Better understanding of the relationship between cognitive functioning and health self-efficacy and neighborhood environment is critical for designing programmatic and policy interventions aimed at supporting proactive aging in older adulthood.

